# Prevention and Management of Infectious Complications in Retrograde Intrarenal Surgery: A Comprehensive Review

**DOI:** 10.7759/cureus.69335

**Published:** 2024-09-13

**Authors:** Sandeep Reddy Ramala, Suresh R Chandak, Hima Swetha Avula, Srinivasulareddy Annareddy

**Affiliations:** 1 General Surgery, Jawaharlal Nehru Medical College, Datta Meghe Institute of Higher Education and Research, Wardha, IND; 2 Pediatrics, Sri Venkateshwara Medical College, Tirupati, IND; 3 Respiratory Medicine, Jawaharlal Nehru Medical College, Datta Meghe Institute of Higher Education and Research, Wardha, IND

**Keywords:** antibiotic prophylaxis, infection prevention, infectious complications, retrograde intrarenal surgery (rirs), urinary tract infections (utis), urosepsis

## Abstract

Retrograde intrarenal surgery (RIRS) is a minimally invasive procedure increasingly used to treat renal stones and other intrarenal pathologies due to its reduced risk of complications, shorter recovery time, and lower postoperative pain compared to more invasive surgical techniques. However, despite its advantages, RIRS is associated with a significant risk of infectious complications, ranging from simple urinary tract infections (UTIs) to severe systemic infections such as urosepsis, which can lead to increased morbidity, prolonged hospitalization, and, in severe cases, mortality. This review aims to summarize the current knowledge on preventing and managing infectious complications associated with RIRS. By exploring the pathophysiology of these infections, identifying patient and procedural risk factors, and evaluating evidence-based strategies for prevention and management, this review provides comprehensive insights into minimizing infection risks in RIRS. A thorough literature review was conducted, examining studies and clinical trials that address the incidence, risk factors, prevention strategies, and management protocols for infections in RIRS. This review also assesses current guidelines from professional organizations and recent infection control technologies and practices advancements. The review identifies multiple risk factors contributing to infections in RIRS, including patient-specific factors such as comorbidities and procedural factors like the duration of surgery and use of instrumentation. Effective prevention strategies include preoperative antibiotic prophylaxis, stringent aseptic techniques during surgery, and careful postoperative monitoring. The review also highlights the importance of a multidisciplinary approach involving urologists, infectious disease specialists, and microbiologists in managing complex cases of infection. Infectious complications remain a significant concern in RIRS, necessitating a comprehensive approach to prevention and management. By adhering to evidence-based guidelines and utilizing a multidisciplinary strategy, healthcare professionals can significantly reduce the incidence of infections, thereby improving patient outcomes and the overall safety of RIRS. Future research should focus on advancing infection control technologies and developing novel prophylactic and therapeutic approaches to further enhance the safety and effectiveness of RIRS.

## Introduction and background

Retrograde intrarenal surgery (RIRS) is a minimally invasive procedure for managing kidney stones and other intrarenal pathologies [1]. This procedure uses a flexible ureteroscope, advanced through the urethra, bladder, and ureter to access the renal pelvis and calyces directly. Once the kidney is reached, stones or other obstructions can be visualized and treated using tools such as lasers for stone fragmentation [2]. The advantages of RIRS over more invasive procedures, like percutaneous nephrolithotomy (PCNL) or open surgery, include reduced postoperative pain, shorter hospital stays, quicker recovery, and fewer complications. These benefits have made RIRS an increasingly preferred choice for treating renal stones, especially those located in the lower pole of the kidney or patients with contraindications for more invasive surgeries [3].

Despite the minimally invasive nature of RIRS, the procedure carries a significant risk of both infectious and non-infectious complications. Infectious complications can range from simple urinary tract infections (UTIs) to more severe systemic infections such as urosepsis, which can be life-threatening if not promptly and effectively managed. The risk of infection during RIRS is influenced by various factors, including the introduction of bacteria during ureteroscope insertion, manipulation of infected stones, prolonged surgical time, and pressurized irrigation fluids, all of which can contribute to bacteremia. Patient-specific factors such as immunosuppression, diabetes, pre-existing infections, and compromised renal function further elevate the susceptibility to postoperative infections. Recent studies estimate that the incidence of post-RIRS infectious complications ranges from 5% to 10%, with urosepsis occurring in approximately 0.3% to 2.5% of cases, depending on the patient population and procedural factors [4].

Non-infectious complications, though less frequent, can also significantly impact outcomes. These may include ureteral injury, bleeding, renal colic due to residual stone fragments, and, in rare cases, perforation of the renal pelvis or ureter, which could necessitate additional interventions. Additionally, prolonged operative times have been associated with a higher likelihood of thermal injury due to the use of energy sources during stone fragmentation, potentially leading to postoperative pain and longer recovery periods. According to recent data, the overall complication rate for RIRS, including infectious and non-infectious events, is between 9% and 15%, underscoring the need for vigilant perioperative management to mitigate these risks [5]. Addressing both infectious and non-infectious risks is critical to improving patient outcomes. This involves optimizing perioperative antibiotic prophylaxis, minimizing operative time, and carefully selecting patients to reduce the likelihood of complications. Furthermore, understanding the pathophysiology, identifying risk factors, and implementing effective preventive measures remain essential for enhancing the safety and efficacy of RIRS [5].

This comprehensive review aims to summarize the current knowledge regarding infectious complications associated with RIRS, explore the mechanisms by which infections occur, identify patient and procedural risk factors, and outline strategies for prevention. The guidelines in this review are based on recommendations from the American Urological Association (AUA) and the European Association of Urology (EAU), emphasizing key infection prevention practices in RIRS. These include preoperative screening for bacteriuria, prophylactic antibiotics tailored to local resistance patterns, adherence to aseptic techniques during surgery, and minimizing operative time and instrument exchanges to reduce contamination risks. Intraoperative measures focus on maintaining a sterile field and minimizing trauma to the urinary tract. At the same time, postoperative care includes continued antibiotic use based on surgical findings, early catheter and stent removal, and vigilant monitoring for infections. By following these evidence-based guidelines, healthcare professionals can significantly reduce the incidence of infectious complications, improve patient outcomes, and optimize the overall effectiveness of RIRS.

## Review

Pathophysiology of infectious complications

Understanding the pathophysiology of infectious complications following RIRS is essential for effective prevention and management. This overview will examine the various types of infections that can arise, the mechanisms responsible for them, and the risk factors contributing to their development [6]. Infectious complications following RIRS can present in multiple forms, with UTIs being the most prevalent. UTIs typically result from bacterial colonization of the urinary tract, leading to symptoms such as dysuria, increased frequency, and urgency [7]. Risk factors for UTIs include pre-existing urinary tract abnormalities, prolonged catheterization, and inadequate antibiotic prophylaxis. Sepsis is another significant infectious complication - a severe systemic response to infection that can occur when bacteria enter the bloodstream, potentially causing multi-organ dysfunction [8]. Sepsis may develop from untreated UTIs or postoperative wound infections and necessitate immediate medical intervention. Although postoperative wound infections are relatively uncommon in RIRS due to their minimally invasive nature, they can still occur, particularly in patients with compromised skin integrity or those undergoing concurrent surgical procedures. Signs of wound infections include redness, swelling, and discharge at the surgical site [9].

**Figure 1:**
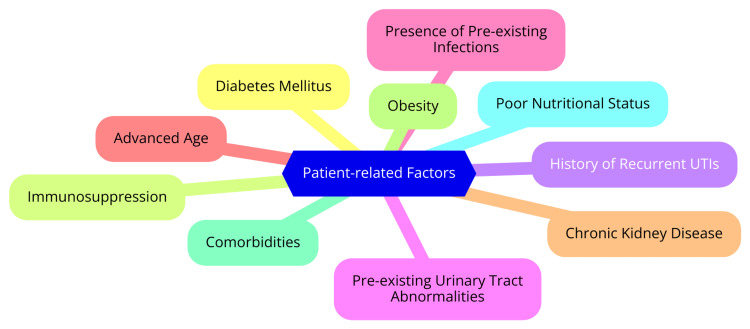
Patient-related risk. Image credit: Sandeep Reddy Ramala.

The development of infections following RIRS primarily involves bacterial entry and proliferation. Bacteria can enter the urinary tract through several routes, including ascending infections, where bacteria from the periurethral area ascend through the urethra into the bladder, resulting in UTIs [10]. In rare instances, hematogenous spread can occur, allowing bacteria to enter the urinary tract through the bloodstream, particularly in immunocompromised patients. Once bacteria infiltrate the urinary tract, they can multiply and cause infection, especially if pre-existing conditions facilitate bacterial growth. Additionally, instrumentation and procedural factors significantly influence the risk of infection. Using instruments during RIRS, such as ureteroscopes and access sheaths, can introduce bacteria into the urinary tract. Factors such as the duration of the procedure, intraoperative trauma to the urethra or bladder, and inadequate sterilization of equipment further elevate the risk of infection [10]. Several risk factors contribute to the likelihood of developing infectious complications following RIRS, which can be categorized into patient-related and procedural factors. Patient-related factors include conditions such as diabetes mellitus, which is associated with impaired immune responses and an increased risk of UTIs. Immunosuppression, whether due to underlying health conditions like HIV or as a consequence of organ transplantation, also heightens susceptibility to infections [11]. Additionally, a history of recurrent UTIs can predispose patients to postoperative infections. On the procedural side, factors such as the duration of surgery are critical; longer surgical times are linked to a higher risk of infection due to prolonged exposure to potential contaminants. Ureteral stents can also be a nidus for bacterial colonization, particularly if left in place for extended periods. Moreover, any breach in sterile technique during the procedure can introduce pathogens, increasing the risk of infection [12]. Patient-related risk factors are illustrated in Figure 1.

Prevention strategies

Preoperative measures are vital in minimizing the risk of infectious complications associated with RIRS. First, patient preparation and screening should prioritize identifying and optimizing modifiable risk factors, such as diabetes and immunosuppression [13]. Screening for active UTIs and treating them before the procedure to prevent postoperative infections is essential. Additionally, prophylactic antibiotics' selection, timing, and duration are critical in infection prevention. Antibiotics should be chosen based on local resistance patterns and tailored to the patient's needs [14]. Ideally, they should be administered within one hour before the incision and continued for 24 to 48 hours post surgery, depending on the clinical scenario. Preoperative hygiene and antiseptics are also crucial components of infection prevention. Patients should be instructed to shower with chlorhexidine gluconate soap the night before and the morning of surgery [15]. This practice helps reduce skin flora, minimizing the risk of contamination during the procedure. Furthermore, proper skin preparation at the surgical site using an appropriate antiseptic agent is essential to creating a sterile surgical environment [16].

During the intraoperative phase, several techniques can help reduce the risk of infections. Foremost, the sterilization of instruments and equipment must adhere to strict protocols to ensure all reusable instruments are properly sterilized [17]. Sterile, single-use devices are encouraged whenever possible to further minimize infection risks. Additionally, minimizing the procedure duration is crucial; optimizing surgical techniques can reduce the time spent in surgery, thereby decreasing exposure to potential contaminants. This includes minimizing the number of instrument exchanges and maintaining a sterile field throughout the procedure. Protective barriers and aseptic techniques are also essential [18]. Surgical teams should wear sterile gowns, gloves, and masks to maintain a sterile environment. Employing sterile drapes to isolate the surgical site further enhances asepsis and helps prevent infections during the procedure [18].

Postoperative care is equally important in preventing infectious complications. Monitoring for early signs of infection should be a priority, with healthcare providers assessing patients for symptoms such as fever, pain, swelling, redness, or purulent discharge at the surgical site. Monitoring urine output and appearance can also provide valuable insights into the patient's recovery and potential complications [9]. The administration and management of postoperative antibiotics should be guided by intraoperative findings and the patient's risk factors. Continuing antibiotics based on culture results and clinical response is essential for effective infection management. Lastly, guidelines for catheter and stent management should be followed closely. Catheters and stents should be removed as soon as clinically appropriate, and patients should be educated on proper care to prevent infections. By implementing these comprehensive prevention strategies throughout the preoperative, intraoperative, and postoperative phases, healthcare providers can significantly reduce the risk of infectious complications in RIRS. This proactive approach leads to improved patient outcomes and reduced healthcare costs associated with complications from kidney stone disease [6]. Prevention strategies for infectious complications in RIRS are shown in Table 1.

**Table 1:** Prevention strategies for infectious complications in retrograde intrarenal surgery (RIRS).

Category	Strategy	Details
Preoperative measures [19]	Patient preparation and screening	Screen for asymptomatic bacteriuria and treat before surgery; assess patient history for risk factors (e.g., diabetes and immunosuppression).
	Prophylactic antibiotics	Administer antibiotics based on culture results and local guidelines, typically given 30–60 minutes before the procedure.
	Preoperative hygiene and antisepsis	Ensure proper hygiene protocols, such as patient bathing with antiseptic soap and using antiseptic solutions for skin preparation.
Intraoperative techniques [20]	Sterilization of instruments and equipment	Follow strict sterilization protocols for all surgical instruments and ensure the operating environment is sterile.
	Minimizing procedural duration	Reduce the length of the surgery to lower the risk of infection by avoiding prolonged exposure and manipulation.
	Use of protective barriers and aseptic techniques	Utilize sterile drapes, gloves, and gowns, and adhere to aseptic techniques to minimize contamination during the procedure.
Postoperative care [21]	Monitoring for early signs of infection	Observe patients for symptoms of infection, such as fever or dysuria, and perform regular check-ups for early detection.
	Postoperative antibiotic administration and management	Continue antibiotics as needed based on intraoperative findings and postoperative assessments; adjust based on culture results.
	Catheter and stent management	Employ appropriate timing for removal of catheters and stents to reduce the risk of infection; use antibiotic-coated stents if indicated.

Management of infectious complications

Managing infectious complications following RIRS necessitates prompt and effective intervention to prevent severe outcomes. A comprehensive clinical assessment involves a detailed history and physical examination to identify signs of infection, such as fever, chills, flank pain, dysuria, or changes in urinary output [4]. Laboratory tests, including urinalysis and urine culture, are crucial for identifying the causative organism and determining its antibiotic susceptibility. Blood tests, such as a complete blood count and blood cultures, are particularly important in cases of suspected sepsis, as they can reveal leukocytosis and guide further management [22]. Imaging studies, such as ultrasound and computed tomography (CT) scans, provide critical information about the urinary tract. Ultrasound is a non-invasive tool that can assess kidney size, detect hydronephrosis, and identify abscesses or other complications. CT scans offer more detailed visualization, particularly useful in complicated cases where initial management is ineffective, to identify the presence of stones, obstruction, or abscess formation [23]. Antibiotic therapy is the cornerstone of treatment, with initial empirical therapy aimed at covering common uropathogens based on local resistance patterns. Once culture results are available, antibiotics should be adjusted to ensure targeted effectiveness against the identified organism. Antibiotic therapy typically ranges from seven to 14 days, depending on the severity of the infection and the patient's clinical response [24]. Early recognition and management are crucial in sepsis or systemic infections. This includes prompt administration of intravenous fluids for resuscitation, broad-spectrum antibiotics, and continuous monitoring of vital signs. In severe cases, admission to an intensive care unit may be necessary for close monitoring and advanced supportive care. If imaging studies indicate the presence of an abscess or significant obstruction, surgical interventions, such as percutaneous drainage or surgical decompression, may be required to relieve the obstruction and drain infected areas [25]. A multidisciplinary approach is essential for effectively managing infectious complications, involving collaboration between urologists, infectious disease specialists, and microbiologists. Regular case reviews and the development of standardized protocols can enhance patient outcomes and streamline care processes. By implementing these strategies, healthcare providers can significantly improve patient outcomes and reduce the risk of severe complications following RIRS [26]. Management strategies for infectious complications following RIRS are detailed in Table 2.

**Table 2:** Management strategies for infectious complications following retrograde intrarenal surgery (RIRS).

Management strategy	Description	Indications	Considerations
Antibiotic therapy [7]	Administration of appropriate antibiotics based on culture and sensitivity results.	First-line treatment for UTIs and bacteremia post-RIRS.	Local antibiograms should guide selection and adjust based on culture results and clinical response.
Supportive care [27]	Includes hydration, pain management, and monitoring of vital signs.	All patients with suspected or confirmed infections.	Monitor for signs of worsening infection or sepsis. Maintain adequate hydration to support renal function.
Management of sepsis [28]	Aggressive fluid resuscitation, vasopressors if needed, and broad-spectrum antibiotics.	Patients showing signs of systemic infection or sepsis.	Follow sepsis protocols, including early identification and management; adjust antibiotics based on cultures.
Surgical intervention [29]	Drainage of abscesses, removal of infected stones, or placement of nephrostomy tubes.	Complicated infections with abscess formation, obstructive uropathy, or infected stones.	Additional imaging may be required to guide intervention; consider the risks versus benefits of further surgery.
Multidisciplinary approach [7]	Involvement of urologists, infectious disease specialists, and intensivists.	Complex cases with high risk of complications or multi-drug resistant organisms.	Collaborative care enhances outcomes, especially in immunocompromised patients or those with comorbid conditions.
Close monitoring and follow-up [4]	Regular clinical assessments, laboratory tests, and imaging as needed.	All patients are recovering from infections post-RIRS.	Frequent reassessment is necessary to detect recurrence or complications and adjust treatment based on clinical progress.

Outcomes and evidence-based practices

Recent studies have highlighted the effectiveness of various preventive measures in reducing infection rates within healthcare settings. Comprehensive infection prevention and control (IPC) strategies have significantly reduced the incidence of healthcare-associated infections (HAIs). For example, during the COVID-19 pandemic, enhanced IPC measures led to a substantial reduction in healthcare-associated respiratory viral infections (HA-RVI) and methicillin-resistant *Staphylococcus aureus* (MRSA) infections [30-32]. One study reported a dramatic decrease in the cumulative incidence of HA-RVI, from 9.69 to 0.83 cases per 10,000 patient days, underscoring the effectiveness of strict adherence to IPC protocols. Different prevention strategies' outcomes vary, highlighting the necessity for tailored approaches [33]. Evidence indicates that fundamental practices, such as hand hygiene, the proper use of personal protective equipment (PPE), and adherence to standard precautions, are essential for reducing infection rates [34]. The Institute of Medicine has noted that appropriate hand hygiene can be more cost-effective than many other interventions in preventing HAIs. Moreover, implementing evidence-based infection control strategies has led to a downward trend in certain HAIs within intensive care units (ICUs) over the past decade, despite ongoing challenges such as increasing antimicrobial resistance and staffing shortages [34]. Professional organizations, including the American Urological Association (AUA) and the European Association of Urology (EAU), have established guidelines to standardize infection control practices across healthcare settings. These guidelines emphasize the importance of IPC measures, including recommendations for preoperative screening, appropriate antibiotic prophylaxis, and the implementation of standard precautions to minimize the risk of infections during surgical procedures [35]. Current evidence supports several best practices for infection prevention. Standard precautions, which include hand hygiene, the use of PPE, and safe handling of medical instruments, are essential for all patient interactions to prevent the spread of infections. In addition to standard precautions, specific transmission-based precautions, such as contact, droplet, and airborne precautions, are necessary for patients with known infections to prevent transmission within healthcare facilities [36]. Continuous education and training for healthcare workers on infection control practices are crucial to maintaining compliance and adapting to new challenges, such as emerging pathogens and evolving healthcare environments [37].

Future directions and research

Advancements in antimicrobial coatings and infection control technologies pave the way for significant improvements in preventing infections associated with medical devices and surgical procedures. Researchers are developing novel antimicrobial coatings that can be applied to various medical instruments, including those used in RIRS [38]. These coatings encompass a range of materials, such as polymeric and organic antiviral substances capable of effectively inactivating viruses. Intrinsically, antimicrobial coatings leverage the material's inherent properties to kill or repel bacteria. Additionally, incorporating metal and metal oxide nanoparticles into these coatings has shown promise in enhancing their antibacterial and antifungal efficacy. Such advancements could reduce the risk of postoperative infections by providing continuous antimicrobial activity on the surfaces of surgical instruments. Emerging technologies, initially developed for decontaminating food contact surfaces, such as antimicrobial bottle coatings and cold plasma treatment, are also being explored for potential applications in medical settings [38]. Beyond improved coatings, developing novel prophylactic and therapeutic agents is another critical focus area. Researchers are investigating the potential of antimicrobial peptides and natural compounds, such as essential oils, for their antibacterial, antiviral, and antifungal properties. These agents represent alternative strategies for infection prevention in surgical contexts, especially for patients at high risk of developing infections. Moreover, antibiotic-based coatings that deliver targeted antimicrobial therapy directly at the site of infection are being explored to enhance infection control without the systemic side effects of traditional antibiotic use. Zwitterionic polymers and other surface-modifying agents are also gaining attention for their ability to prevent bacterial attachment and biofilm formation, significantly contributing to postoperative infections [39].

Despite these advancements, there remain notable gaps in our current knowledge and practice regarding the implementation and effectiveness of antimicrobial technologies. One key area for further investigation is optimizing coating composition, stability, and long-term efficacy to ensure sustained antimicrobial activity throughout use. Additionally, understanding the impact of these coatings on the host immune response and the potential for developing resistance is crucial for their safe application in clinical settings. Translating promising laboratory findings into clinically relevant applications and large-scale manufacturing processes presents another challenge requiring focused research efforts [39]. To specifically address the infectious complications associated with RIRS, future studies should consider several avenues. One potential research area is the development of antimicrobial coatings specifically designed for ureteroscopes and other instruments used in RIRS, which could significantly reduce the risk of device-related infections. Another promising approach involves investigating antimicrobial hydrogels or other local delivery systems that provide sustained antibiotic prophylaxis directly at the surgical site, minimizing the risk of infection while avoiding systemic antibiotic exposure [40]. Additionally, exploring innovative treatment modalities, such as antimicrobial photodynamic therapy, could offer new options for eradicating persistent infections in the urinary tract. By focusing on these emerging technologies and addressing key research gaps, the field of infection prevention and management in RIRS can continue to evolve, ultimately leading to improved patient outcomes and reduced complications [41]. Future directions and research in preventing and managing infectious complications in RIRS are outlined in Table 3.

**Table 3:** Future directions and research in preventing and managing infectious complications in retrograde intrarenal surgery (RIRS).

Future direction	Description	Potential impact
Emerging antimicrobial technologies [42]	Developing and applying new antimicrobial coatings and materials for ureteroscopes and other surgical instruments.	Could reduce the bacterial load and prevent infections, improving overall patient safety.
Novel prophylactic agents [43]	Research into new antibiotics, antiseptics, and other agents that can be used preoperatively to prevent infections.	Enhances the effectiveness of infection prevention, especially in patients with antibiotic resistance.
Personalized medicine approaches [44]	To tailor prophylactic strategies, utilize patient-specific risk factors, such as genetic predisposition to infection or immune response.	Enables targeted infection prevention strategies, potentially reducing unnecessary antibiotic use.
Minimally invasive techniques [45]	Innovations in RIRS techniques that minimize mucosal trauma and reduce operative time, thereby lowering infection risks.	Reduces the incidence of infection by minimizing procedural risks associated with traditional methods.
Real-time monitoring technologies [45]	Development of sensors and monitoring systems that provide real-time data on potential infections during and after surgery.	Allows for immediate intervention, potentially reducing the severity and duration of infections.
Comparative effectiveness research [46]	Studies comparing the effectiveness of different infection prevention and management strategies, including antibiotic regimens and surgical techniques.	Identifies the most effective approaches, leading to standardized best practices in clinical settings.
Long-term outcome studies [47]	Research to evaluate the long-term outcomes of different infection management strategies in RIRS, including quality of life and recurrence rates.	Provides data on the long-term efficacy of different management approaches, guiding future protocols.
Interdisciplinary collaboration [48]	Encouraging collaboration between urologists, infectious disease specialists, microbiologists, and researchers to develop comprehensive infection prevention strategies.	Promotes a holistic approach to infection prevention, integrating multiple perspectives and expertise.

## Conclusions

Infectious complications in RIRS remain a significant concern despite the procedure’s minimally invasive nature and numerous benefits. These complications can range from minor UTIs to severe, life-threatening sepsis, underscoring the need for a thorough understanding of the risk factors and effective preventive strategies. By comprehensively reviewing the pathophysiology, patient-specific and procedural risk factors, and current prevention and management practices, this review highlights the importance of a multi-faceted approach to reducing infection risks in RIRS. Evidence-based strategies, such as the judicious use of prophylactic antibiotics, meticulous aseptic techniques, and vigilant postoperative monitoring, are crucial in minimizing the incidence of infections and improving patient outcomes. Furthermore, future research should focus on refining these strategies and developing innovative approaches to further enhance the safety and efficacy of RIRS. By adhering to the guidelines and recommendations provided in this review, clinicians can better safeguard against infectious complications, ensuring that the advantages of RIRS are fully realized for their patients.
